# Efficacy and safety of glucocorticoids use in patients with COVID-19: a systematic review and network meta‑analysis

**DOI:** 10.1186/s12879-023-08874-w

**Published:** 2023-12-20

**Authors:** Qiaolan He, Chen Wang, Yingqin Wang, Guannan Chen, Yue Zhou, Yuanyuan Wu, Ming Zhong

**Affiliations:** 1 Department of Critical Care Medicine, Zhongshan Hospital, Fudan University, 180 Fenglin Road, Shanghai, 200030 China; 2 Shanghai Key Laboratory of Lung Inflammation and Injury, 180 Fenglin Road, Shanghai, 200030 China; 3 Shanghai Institute of Infectious Disease and Biosecurity, School of Public Health, Fudan University, Shanghai, 200030 China

**Keywords:** COVID-19, Glucocorticoids, Network meta-analysis

## Abstract

**Background:**

Currently, some meta-analyses on COVID-19 have suggested that glucocorticoids use can reduce the mortality rate of COVID-19 patients, utilization rate of invasive ventilation, and improve the prognosis of patients. However, optimal regimen and dosages of glucocorticoid remain unclear. Therefore, the purpose of this network meta-analysis is to analyze the efficacy and safety of glucocorticoids in treating COVID-19 at regimens.

**Methods:**

This meta-analysis retrieved randomized controlled trials from the earliest records to December 30, 2022, published in PubMed, Embase, Cochrane Library, CNKI Database and Wanfang Database, which compared glucocorticoids with placebos for their efficacy and safety in the treatment of COVID-19, Effects of different treatment regimens, types and dosages (high-dose methylprednisolone, very high-dose methylprednisolone, Pulse therapy methylprednisolone, medium-dose hydrocortisone, high-dose hydrocortisone, high-dose dexamethasone, very high-dose dexamethasone and placebo) on 28-day all-caused hospitalization mortality, hospitalization duration, mechanical ventilation requirement, ICU admission and safety outcome were compared.

**Results:**

In this network meta-analysis, a total of 10,544 patients from 19 randomized controlled trials were finally included, involving a total of 9 glucocorticoid treatment regimens of different types and dosages. According to the analysis results, the 28-day all-cause mortality rate was the lowest in the treatment with pulse therapy methylprednisolone (OR 0.08, 95% CI 0.02, 0.42), but the use of high-dose methylprednisolone (OR 0.85, 95% CI 0.59, 1.22), very high-dose dexamethasone (OR 0.95, 95% CI 0.67, 1.35), high-dose hydrocortisone (OR 0.64, 95% CI 0.34, 1.22), medium-dose hydrocortisone (OR 0.80, 95% CI 0.49, 1.31) showed no benefit in prolonging the 28-day survival of patient. Compared with placebo, the treatment with very high-dose methylprednisolone (MD = -3.09;95%CI: -4.10, -2.08) had the shortest length of hospital stay, while high-dose dexamethasone (MD = -1.55;95%CI: -3.13,0.03) and very high-dose dexamethasone (MD = -1.06;95%CI: -2.78,0.67) did not benefit patients in terms of length of stay.

**Conclusions:**

Considering the available evidence, this network meta‑analysis suggests that the prognostic impact of glucocorticoids in patients with COVID-19 may depend on the regimens of glucocorticoids. It is suggested that pulse therapy methylprednisolone is associated with lower 28-day all-cause mortality, very high-dose methylprednisolone had the shortest length of hospital stay in patients with COVID-19.

**Trial registration:**

PROSPERO CRD42022350407 (22/08/2022).

**Supplementary Information:**

The online version contains supplementary material available at 10.1186/s12879-023-08874-w.

## Introduction

SARS-Cov-2 was first discovered in Wuhan, China in 2019 [1]. COVID-19, caused by SARS-Cov-2 [2], has been declared as a global pandemic by world health organization (WHO) in March 2020. The main clinical manifestations of COVID-19 are fever, dry cough and fatigue, with a small number of patients accompanied by nasal congestion, runny nose, sore throat and diarrhea [3], patients with severe novel coronavirus pneumonia are characterized by a severe cytokine storm, in which the overproduction of pro-inflammatory cytokines leads to increased vascular permeability and multiple organ failure [4], poses severe challenges to not only to human health, but also global health care system [5, 6].

As effective anti-inflammatory drugs, glucocorticoids are often used as adjuvant treatment of viral pneumonias and ARDS treatments [7], such as severe acute respiratory syndrome (SARS) [8], middle east respiratory syndrome (MERS) [9], etc. National Institutes of Health in the United States have included glucocorticoids as a treatment for COVID-19 patient [10]. Glucocorticoid bind to the glucocorticoid receptors, thus affects many physiological pathways, including metabolism, cell apoptosis, and benefits COVID-19 patients through its immunosuppressive action [11]. Some recent studies suggest that the use of glucocorticoids can effectively reduce the mortality, increase ventilator-free days and improve the prognosis of COVID-19 patients [12, 13]. However, the glucocorticoid regimen and dosage used in those studies are different, so the optimal glucocorticoid regimen for COVID-19 patients remains unknown. Moreover, side effects of glucocorticoids, including hyperglycemia, electrolyte disorders, and water and sodium retention, and so on, make the safety and efficacy of their treatment of COVID-19 still controversial.

This network meta-analysis focuses on whether glucocorticoid therapy can improve the prognosis of COVID-19 patients, to find the optimal glucocorticoid regimen, so as to provide evidence for the clinical use of glucocorticoids in COVID-19 patients.

## Methods

### Protocol and search strategy

The study protocol of this network meta-analysis was registered in the International Prospective Register of Systematic Reviews (PROSPERO) (CRD42022350407) with basic principles of data extraction and the analysis method, the literature search results are reported according to the guidelines of the Preferred Reporting Items for Systematic Reviews and Meta-Analyses statement (PRISMA) for NMA [14] (PRISMA checklist were provided in Additional file 2).

The retrieval languages of this network meta-analysis were Chinese and English, databases including PubMed, Web of Science, Cochrane Library, China National Knowledge Infrastructure Database (CNKI database), Wanfang Database, China Biology Medicine disc(CBMdisc) were searched for published randomized controlled trials. The retrieval period was from the establishment of the database to November 1, 2022. Medical Subject heading (MeSH) terms were used, including COVID-19, glucocorticoid, steroids, etc., while other keywords were limited to title and abstract (details of search strategies were provided in Additional file 1).

### Study selection and data extraction

Only published randomized controlled trials of glucocorticoids for the treatment of COVID-19 were included, excluding studies including case-control studies, cohort studies, etc. Inclusion criteria included: adults(age ≥ 18 years old), confirmed COVID-19 and willingness to provide informed consent. Exclusion criteria included foreseeable and inevitable death, pregnancy, breast-feeding, and use of glucocorticoids for other needs. Full inclusion and exclusion criteria in the appendix (Additional file 1).

Articles included in this network meta-analysis was retrieved and identified by two authors (QH and CW). After full-text review, for articles that met inclusion criteria, patient characteristics, interventions, controls, and outcomes were extracted using Excel, opinions of a third author (MZ) were solicited if necessary.

Based on the literature retrieved, this network meta-analysis has divided glucocorticoid regimens into nine groups [15, 16]: pulse therapy methylprednisolone(> 200 mg/day), very high-dose methylprednisolone(> 80 mg /day, but ≤ 200 mg/day), high-dose methylprednisolone(> 24 mg /day, but ≤ 80 mg/day), very high-dose dexamethasone(> 12 mg /day, but ≤ 37.5 mg/day), high-dose dexamethasone(> 6 mg /day, but ≤ 12 mg/day), medium-dose dexamethasone(> 1.125 mg /day, but ≤ 6 mg/day), high-dose hydrocortisone(> 120 mg /day, but ≤ 400 mg/day), medium-dose hydrocortisone(> 30 mg /day, but ≤ 120 mg/day) and no glucocorticoid use.

### Quality assessment

The risk of bias was assessed by the Cochrane Handbook for Systematic Reviews of Interventions [17, 18], and was assessed independently by two investigators. The evaluation contents including randomization bias, implementation of distribution concealment scheme, blind implementation; integrity of the result data, selective reporting bias and other sources of bias.

### Outcome measures and definitions

The primary outcome of this network meta-analysis is all-cause mortality at 28 days, the secondary outcome is hospitalization duration, the utilization and duration of invasive mechanical ventilation, intensive care unit admission and duration and safety outcome.

### Data analysis

All statistical analyses of this review were performed in STATA, version 17.0 (Stata Corporation, College Station, TX, USA), using frequentist framework. Relative odds ratio (OR) and 95% credible intervals were used as the effect indicators of binary outcome. For continuous variables, mean difference (MD) and 95% credible intervals were used. The level of significance for all analyses was *p* < 0.05, the heterogeneity of the included studies was evaluated by heterogeneity parameter tau-square (τ^2^). When *P* > 0.05 and τ^2^ ≤ 50%, the heterogeneity of the study was small, and the fixed effect model was used. On the contrary, if *P* < 0.05 and τ^2^ > 50%, the random effects model was used. The surface under the cumulative ranking curve (SUCRA) of each intervention was used to reflect the efficacy of different glucocorticoid treatment regimens. The closer it was to 100%, the more likely it was that the treatment regimen had the optimal efficacy. The funnel plot was drawn to determine whether there were publication bias or small sample effect. For studies that only reported the interquartile range and median, we used the methods that were introduced by literature to estimate the mean and standard deviation [19, 20].

## Results

### Study selection

**Fig. 1:**
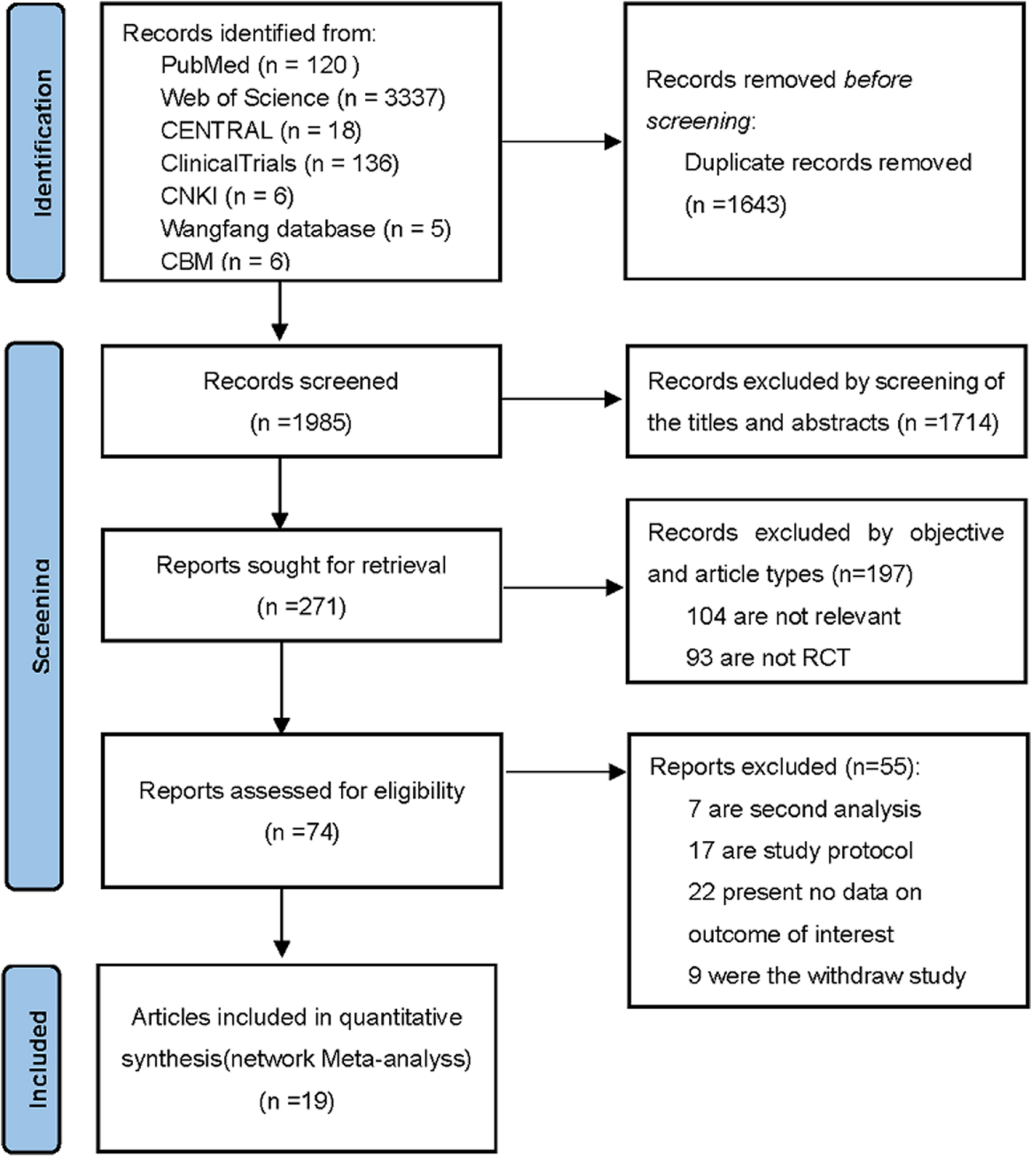
Flow diagram of searching processes

The selection process of included studies selection is shown in Fig. 1. A total of 3877 records were retrieved from PubMed, Embase, Cochrane Library, CNKI full-text database, Wanfang Database, CBMdisc and other sources. After removing duplicate literatures and further screening by reading their titles and abstracts, 1643 articles were excluded. After screening of the titles and abstracts, 1714 articles were excluded. A total of 271 articles were retrieved and under full-text reading and 74 of them were assessed for eligibility. Finally, 19 articles were included for this network meta-analysis.

### Quality assessment

**Fig. 2:**
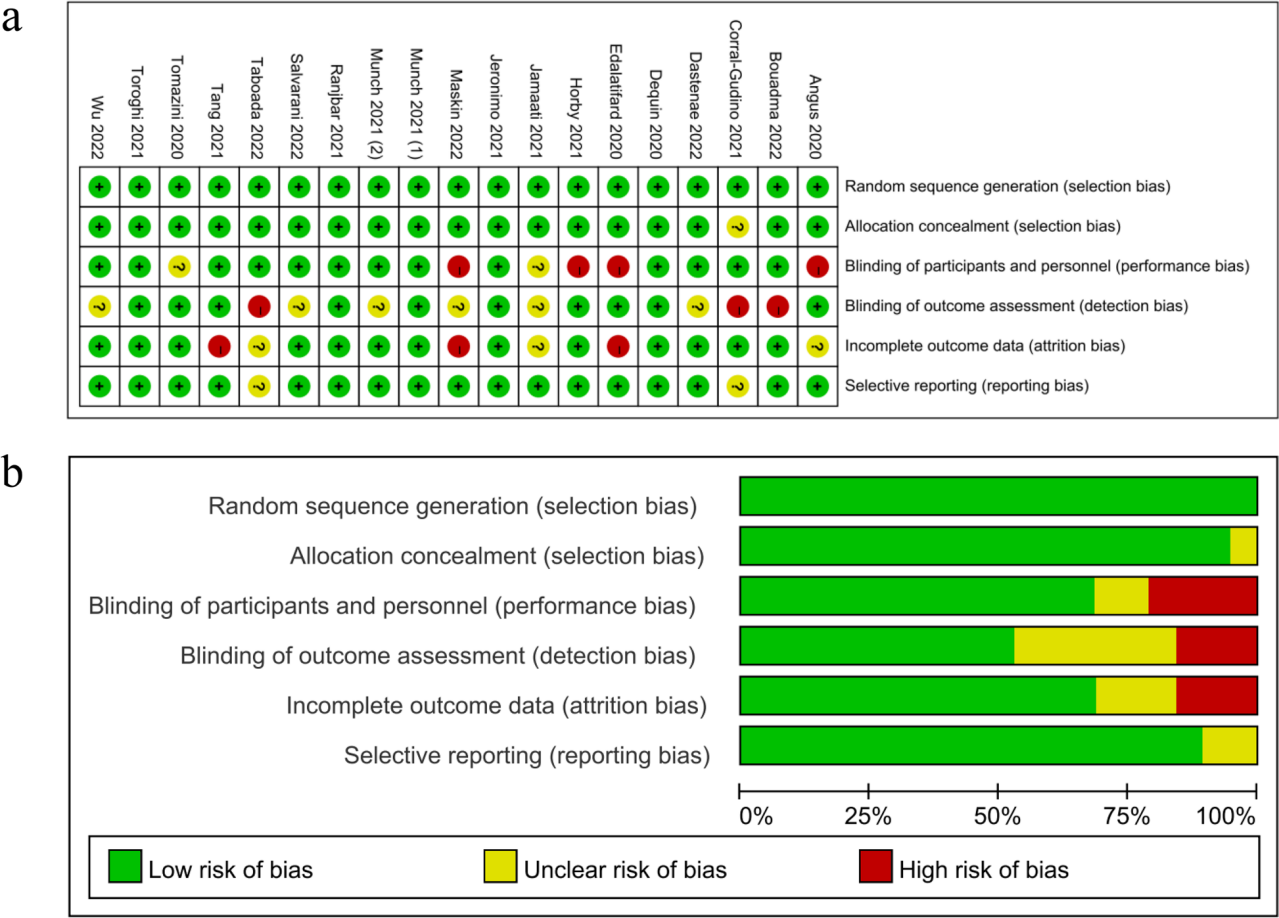
The quality assessment of included randomized controlled trials. **a** Risk of bias summary (Green circles represent “low risk of bias”, yellow circles represent “unclear risk of bias”, red circles represent “high risk of bias”). **b** Risk of bias graph

The quality of included 19 randomized controlled trials were assessed by the Cochrane risk of bias tool and showed by RevMan 5.4 software in Fig. 2. Five studies were considered to have a low risk of bias [21–25], while another 6 studies were assessed as having unclear risk of bias [13, 26–30]. In addition, 8 RCTs [12, 31–37] were considered to have a high risk of bias because of their performance bias, detection bias and attrition bias.

### Study characteristics

A total of 19 randomized controlled trials were included in this meta-analysis. Eighteen of them were two-arm trials [12, 13, 22–37] and one was a three-arm trial [21]. 10,544 patients with COVID-19 were included, with an average age of 61.47 years old, including 35.80% females and 64.20% males. The 28-day all-cause mortality was reported in 16 articles [12, 13, 22–26, 28–35, 37], 9 regimens and dosages of glucocorticoid were involved in the included study; 10 of them reported the length of stay [21, 22, 25, 26, 28, 29, 31–33, 36], while 6 each reported mechanical ventilation requirement [12, 21, 22, 24, 29, 32] and ICU admission [21, 27, 28, 31, 32, 35], the basic characteristics of the included study were shown in Table 1.

**Table 1:** Details of included studies

**Author Year** **Country**	**Sample size**	**Intervention: Control**	**Inclusion criteria**	**Regimen of Corticosteroid**	**Control intervention**		**Planned primary Outcome**	**Time Course**
Edalatifard 2020 [34] Iran	62	34:28	Confirmed COVID-19 with SpO2 < 90%, elevated CRP and IL-6 without ventilator and intubation	Methylprednisolone (IV) 250 mg/d for 3 days	Standard care		The time of clinical improvement or death	20 April 2020–20 June 2020
Corral-gudino 2021 [35] Spain	64	35:29	Adult COVID-19 patients, Symptom duration > 7 days, with evidence of systemic inflammatory response	Methylprednisolone (IV) 40 mg bid for 3 days followed by 20 mg BID for 3 days	Standard care		Composite of death, ICU admission, requirement for NIMV.	April 2020–June 2020
Tang 2021 [31] China	86	43:43	Adult patients with COVID-19 pneumonia who were admitted to the general ward	Methylprednisolone (IV) 1 mg/kg/day for 7 days	Placebo		Clinical deterioration in 14 days	19 February 2020–31 March 2020
Ranjbar 2021 [22] Iran	93	47:46	Hospitalized adult patients, with SpO2 < 92 in room air.	Methylprednisolone (IV) 2 mg/kg/day	Dexamethasone (IV) 6 mg/kg/day		All-cause mortality at 28 days, clinical status at day 5, 10.	August 2020-November 2020
Jeronimo 2021 [25] Brazil	416	209:207	Hospitalized adult patients either had SpO2 ≤ 94% with room air, or required IMV	Methylprednisolone (IV) 0.5 mg/kg BID for 5 days	Placebo		28-day mortality	18 April 2020–16 June 2020
Dequin 2020 [24] France	149	76:73	Confirmed or suspected COVID-19 with acute respiratory failure	Hydrocortisone (IV) 200 mg/d until day 7, then 100 mg/d for 4 days and 50 mg/d for 3 days	Placebo		Treatment failure on day 21	7 March 2020–1 June 2020
Angus 2020 [37] International	379	283:101	Adult patients with suspected or confirmed COVID-19	Hydrocortisone (IV) 50 mg QID for 7 days; while in shock for up to 28 days;	Standard care		Organ support–free days within 21 days	9 March 2020–17 June 2020
Munch 2021 [23] Denmark	30	16:14	Hospitalized adult patients with confirmed SARS-CoV-2 infection and severe hypoxia	Hydrocortisone (IV) 200 mg/day for 7 days	Placebo		Days alive without life support	15 April 2020–3 September 2020
Jamaati 2021 [29] Iran	50	25:25	Adult patients with COVID-19 pneumonia, PaO2/FiO2 between 100 and 300	Dexamethasone (IV) 20 mg/day from day 1–5, then 10 mg/day until day 10	Standard Care		Need for IMV and death rate.	March 2020-
Tomazini 2021 [13] Brazil	299	151:148	Adult patients had confirmed or suspected COVID-19, receiving mechanical ventilation within 48 h	Dexamethasone (IV) 20 mg/day for 5 days, followed by 10 mg/day for additional 5 days or until ICU discharge	Standard care		Ventilator-free days during the first 28 days	17 April 2020–23 June 2020
Munch 2021 [30] Europe, India	1000	503:497	Hospitalized adult COVID-19 patients, required supplementary oxygen > 10 L/min, NIMV, CPAP or IMV	Dexamethasone (IV) 12 mg/day for 10 days	Dexamethasone (IV) 6 mg/ day for 10 days		Days alive without life support at 28 days	27 August 2020–20 May 2021
Horby 2021 [12] United Kingdom	6425	2104:4321	Adult patients who were hospitalized with COVID-19	Dexamethasone (Oral/IV) 6 mg/day for 10 days	Standard care		28-day mortality	19 March 2020–8 June 2020
Maskin 2022 [33] Argentina	100	49:51	Adult patients with confirmed COVID-19-related ARDS	Dexamethasone (IV) 16 mg/day for 5 days, then 8 mg/day for 5 days	Dexamethasone (IV) 6 mg/day for 10 days		Ventilator-free days during 28 days	17 June 2020–27 March 2021
Bouadma 2022 [36] France	546	270:276	Adults with AHRF admitted to ICU for confirmed or suspected COVID-19	Dexamethasone (IV) 20 mg/day on days 1–5 then 10 mg/d on days 6–10	Dexamethasone (IV) 6 mg/day for 10 days		all-cause mortality, assessed at day 60	10 April 10–17 September 2020
Taboada 2021 [32] Spain	200	98:102	Adult patients with confirmed COVID-19, receiving supplemental oxygen	Dexamethasone (IV) 20 mg/day for 5 days, followed by 10 mg/day for 5 days	Dexamethasone (IV) 6 mg/day for 10 days		Clinical worsening within 11 days	15 January 2021–26 May 2021
Dastenae 2022 [28] Iran	143	73:70	All patients with COVID-19 who tested positive by RT-PCR test	Methylprednisolone (IV) 60 mg/day in two divided doses	Dexamethasone (IV) 8 mg/day		Duration of hospitalization	April 2021-June 2021
Salvarani 2022 [27] Italy	304	152:152	Adult patients with COVID-19 infection, requiring supplemental oxygen	Methylprednisolone (IV) 1 g/day for 3 days	Dexamethasone (IV) 6 mg/day for 10 days		Duration of hospitalization	21 December 2020–10 March 2021
Wu 2022 [26] United State	110	55:55	Adult patients with PCR-confirmed COVID-19, needing supplemental oxygen	Dexamethasone (IV) 20 mg/day for 5 days, then 10 mg/day for 5 days	Dexamethasone (IV) 6 mg/day for 10 days		Clinical improvement at day 28	January 2021- December 2021
**Author Year** **Country**	**Sample size**	**Intervention: Control**	**Inclusion criteria**	**Regimen of Corticosteroid**			**Planned primary Outcome**	**Time course**
Toroghi 2021 [21] Iran	144	48:48:48	Hospitalized adult COVID-19 patients, required supplementary oxygen	Dexamethasone(IV) 8 mg TID for 10 days	Dexamethasone(IV) 8 mg BID for 10 days	Dexamethasone(IV) 8 mg/day for 10 days	Clinical response	26 October 2020–25 January 2021

***Abbreviations***: *SpO2* Pulse oxygen saturation, *PaO2/FiO2* Partial pressure of oxygen/ fraction of inspired oxygen, *IV* Intravenous injection, *BID* Bis in die, *NIMV* Non-invasive mechanical ventilation, *QID* Quarter in die, *IMV* Invasive mechanical ventilation, *CPAP* Continuous positive airway pressure, *ARDS* Acute respiratory distress syndrome, *AHRF* Acute hypoxemic respiratory failure, *ICU* Intensive care unit, *TID* Ter in die, *RT-PCR* Reverse transcription–PCR

### Hospital mortality

**Fig. 3:**
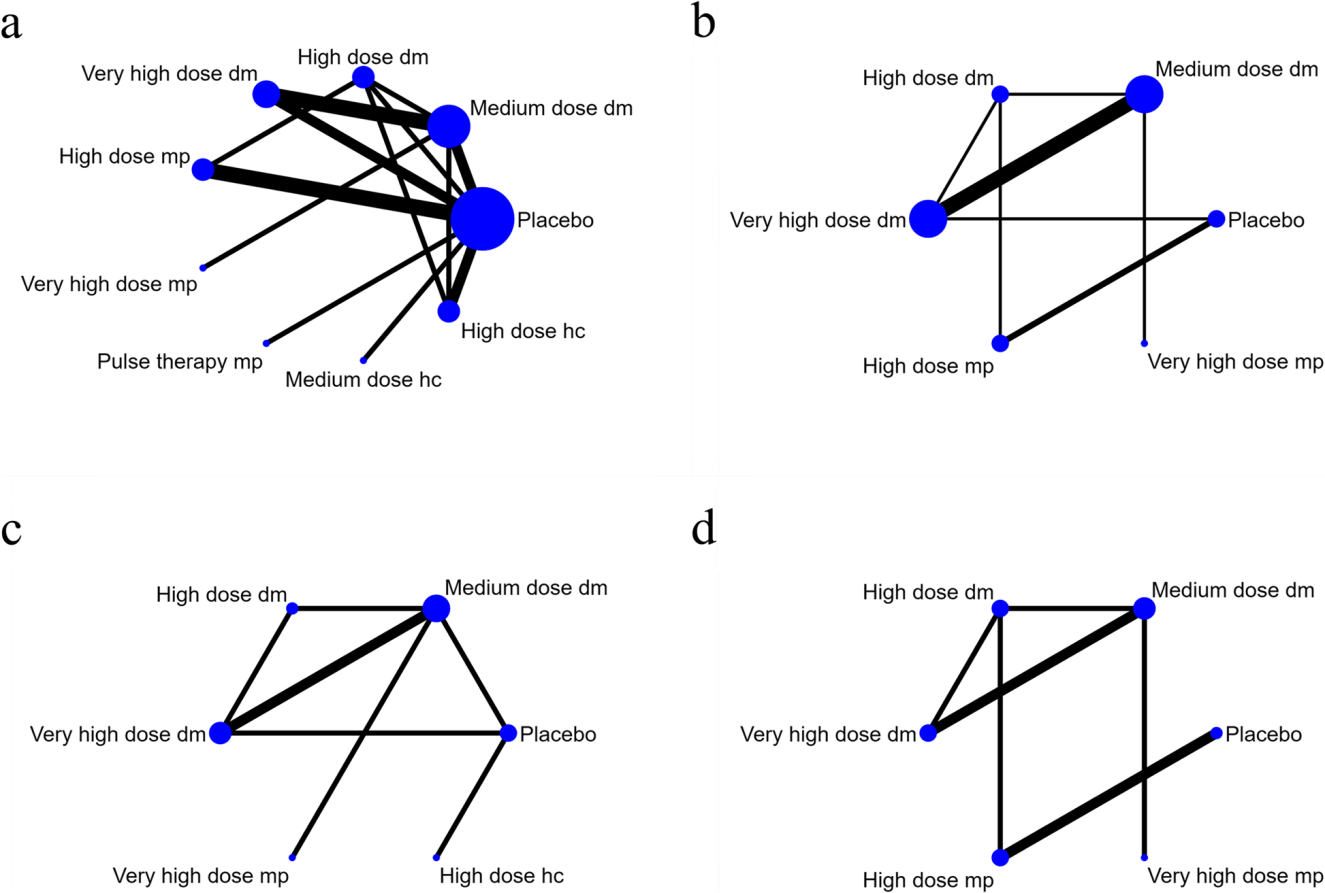
Network plot of different glucocorticoid regimens. **a** 28-day all-cause mortality; **b** Hospitalization duration; **c** Mechanical ventilation requirement; **d** ICU admission. Abbreviations: dm: dexamethasone, mp: methylprednisolone, hc: hydrocortisone

Sixteen articles have reported 28-day all-cause mortality [12, 13, 22–26, 28–35, 37] (*n* = 9536), and their network plots have shown in Fig. 3a. Each node indicates a treatment strategy. The edge represented the number of direct comparisons between two different dosage and regimen of glucocorticoid.

**Fig. 4:**
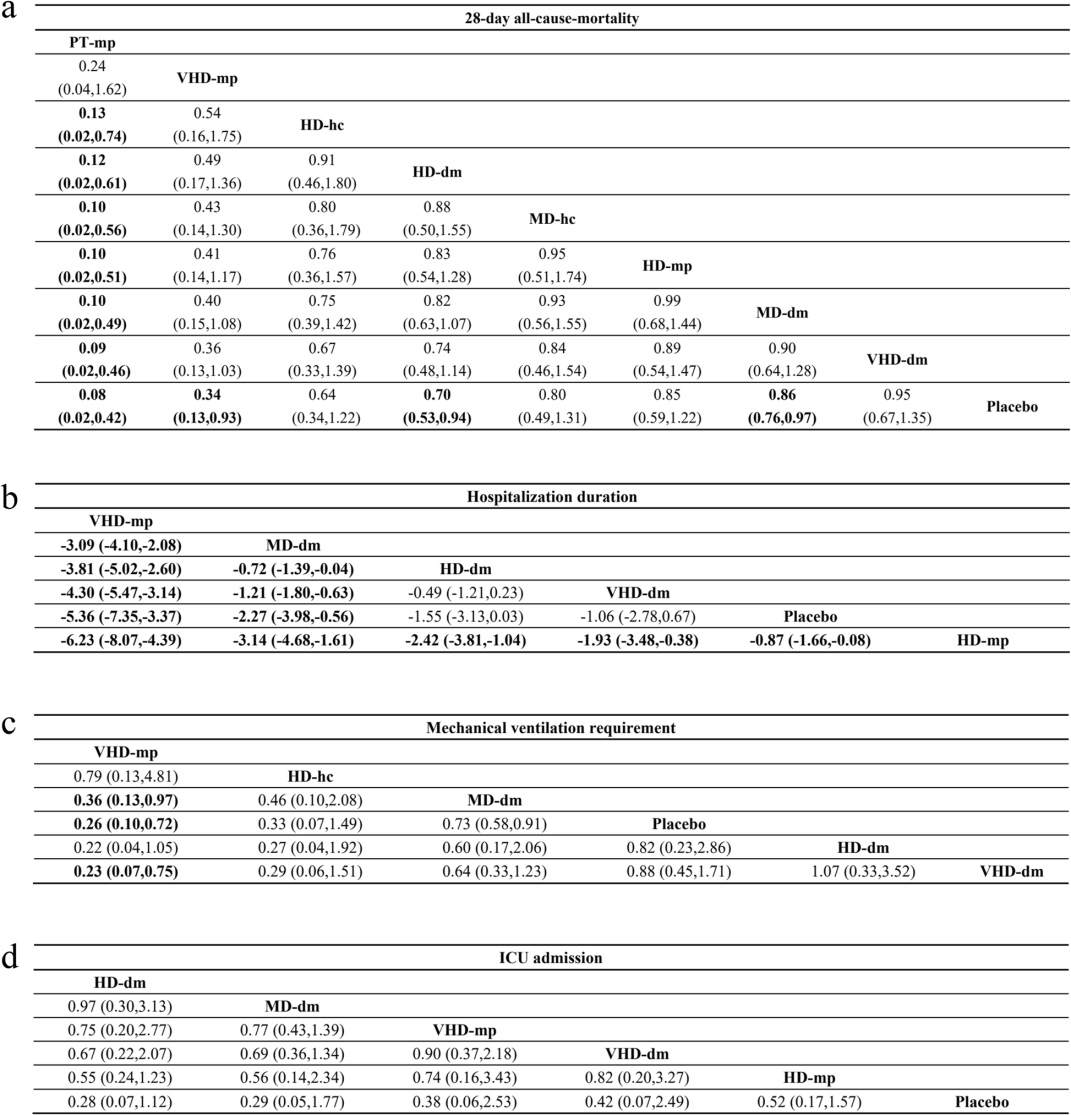
The results of the network meta-analysis. **A** 28-day all-cause mortality; **B** Hospitalization duration; **C** Mechanical ventilation requirement; **D** ICU admission. Abbreviations: MD: medium-dose, HD: high-dose, VHD: very high-dose, PT: pulse therapy

Network meta-analysis showed that, compared with other treatment regimens, pulse therapy methylprednisolone (PT-mp) significantly reduced patient 28-day all-cause mortality, except for very high-dose of methylprednisolone (VHD-mp) (OR 0.24, 95% CI 0.04, 1.62); compared with placebo, half of the treatment regimens can reduce 28-day all-cause mortality in patients with COVID-19, including PT-mp (OR 0.08, 95% CI 0.02, 0.42), VHD-mp (OR 0.34, 95% CI 0.13, 0.93), high-dose dexamethasone (HD-dm) (OR 0.70, 95% CI 0.53, 0.94) and medium-dose dexamethasone (MD-dm) (OR 0.86, 95% CI 0.76, 0.97). There was no significant difference in 28-day all-cause mortality among patients treated with other glucocorticoids regimens and dosages (Fig. 4a).

**Fig. 5:**
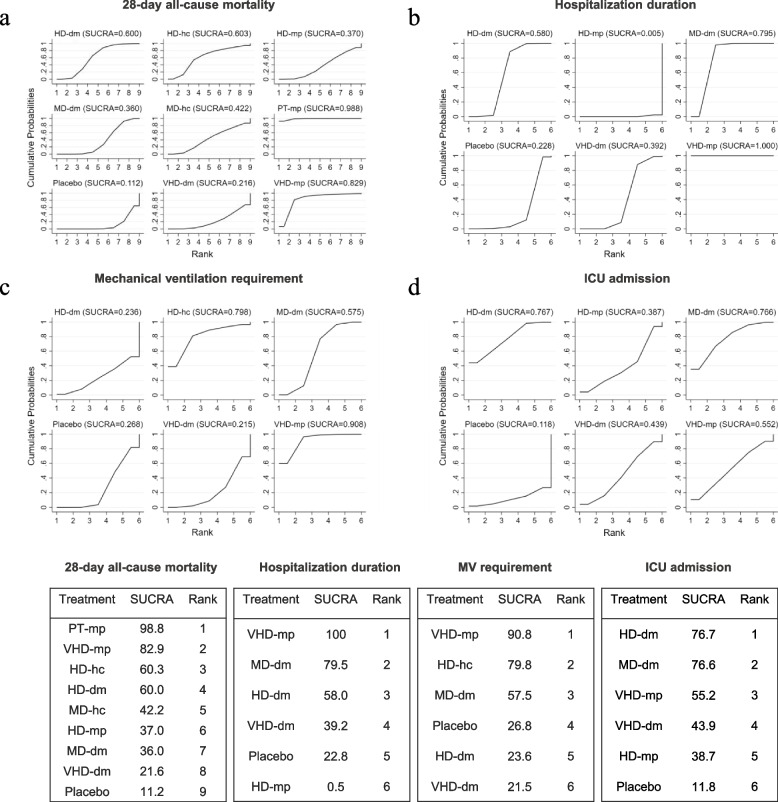
SUCRA ranking charts of different regimen of glucocorticoid. **A** 28-day all-cause mortality; **B** Hospitalization duration; **C** Mechanical ventilation requirement; **D** ICU admission

By analyzing the data of the included articles, the effectiveness of different doses and types of glucocorticoids in reducing 28-day all-cause mortality in patients with COVID-19 is ranked as follows: PT-mp (SUCRA = 98.8%) > VHD-mp (SUCRA = 82.9%) > high-dose hydrocortisone (HD-hc) (SUCRA = 60.3%) > HD-dm (SUCRA = 60%) > medium-dose hydrocortisone (MD-hc) (SUCRA = 42.2%) > high-dose methylprednisolone (HD-mp) (SUCRA = 37%) > MD-dm (SUCRA = 36%) > very high-dose dexamethasone (VHD-dm) (SUCRA = 21.6%) > placebo(SUCRA = 11.2%) (Fig. 5a). There are no comparisons with statistically significant inconsistencies were observed in the node-splitting model.

To assess publication bias, we performed funnel plot analyses of OR and SE (log [OR]) for 28-day all-cause mortality of 9 glucocorticoid regimens. The distribution on both sides of the funnel plot is basically symmetrical, and most of them are concentrated in the middle and upper part of the funnel plot, indicating that there is less possibility of small sample effect or publication bias (Fig. S2a).

### Secondary outcome

Ten articles [21, 22, 25, 26, 28, 29, 31–33, 36] (*n* = 1696) that have been included reported the hospitalization duration of COVID-19 patients, 6 reported mechanical ventilation requirement [12, 21, 22, 24, 29, 32] (*n* = 6926), ICU admission [21, 27, 28, 31, 32, 35](*n* = 921), and their network plots have shown in Fig. 3b, c and d. Mechanical ventilation duration was reported in 3 studies [13, 21, 32] (*n* = 632) and ICU duration was reported in 5 studies [21, 29, 32, 33, 36] (*n* = 951).

#### Hospitalization duration

Compare with other treatments, very high-dose methylprednisolone significantly reduced the length of hospital duration of COVID-19 patients. Hospitalization duration in patient using MD-dm treatment regimen was shorter than other treatment regimen, except for VHD-mp (MD = 3.09;95%CI: 2.08, 4.10); compared with placebo, only VHD-mp (MD = -5.36;95%CI: -7.35, -3.37) and MD-dm (MD = -2.27;95%CI: -3.98, -0.56) could reduce hospitalization duration, and all treatment regimens were better than high-dose methylprednisolone. There was no significant difference among patients receiving other glucocorticoid regimens (Fig. 4b).

SUCRA graph indicated the ranking of 6 glucocorticoid regimens in shortening the length of hospital stay: VHD-mp (SUCRA = 100%) > MD-dm (SUCRA = 79.5%) > HD-dm (SUCRA = 58.0%) > VHD-dm (SUCRA = 39.2%) > placebo(SUCRA = 22.8%) > HD-mp (SUCRA = 0.50%) (Fig. 5b). There are no comparisons with statistically significant inconsistencies were observed in the node-splitting model.

#### Mechanical ventilation requirement

In terms of the need for mechanical ventilation, VHD-mp (OR 0.26, 95% CI 0.10, 0.72) and MD-dm (OR 0.73, 95% CI 0.58, 0.91) reduce mechanical ventilation requirement compared to placebo, VHD-mp reduced the probability of intubation better than MD-dm (OR 0.36, 95% CI 0.13, 0.97). In addition, it is also superior to VHD-dm (OR 0.23, 95% CI 0.07, 0.75). The remaining glucocorticoids showed no significant difference in reducing the need for intubation (Fig. 4c). The SUCRA graph is sorted as follows: VHD-mp (SUCRA = 90.8%) > HD-hc (SUCRA = 79.8%) > MD-dm (SUCRA = 57.5%) > placebo (SUCRA = 26.8%) > HD-dm (SUCRA = 23.6%) > VHD-dm (SUCRA = 21.5%) (Fig. 5c). There are no comparisons with statistically significant inconsistencies were observed in the node-splitting model.

#### Other outcomes

We also conducted network meta-analysis of mechanical ventilation duration, ICU admission and ICU duration. Their network plots have shown in Fig. S1. For mechanical ventilation duration, we found that, neither HD-dm (MD = 0.40;95%CI: -0.15, 0.95), nor VHD-dm (MD = 0.40;95%CI: -0.96, -0.16) can shorten the duration of mechanical ventilation in patients with COVID-19. The MD-dm significantly increased the length of mechanical ventilation (MD = 4.63;95%CI: 3.02, 6.23) (Fig. S3a). For ICU admission and length of stay in the ICU, glucocorticoid regimens did not reduce the rate of admission or length of stay in the ICU compared with placebo (Figs. 4d and 5d, S3b). SUCRA graph were shown in Fig. S4.

### Safety outcomes

A total of 9 articles [13, 21, 23, 24, 27, 30, 34, 36, 37] (*n* = 2881) reported serious adverse effects caused by different treatment regimens, including 8 glucocorticoid regimens as follows: VHD-MP, HD-dm, VHD-dm, MD-dm, PT-mp, placebo, MD-hc, HD-hc, however, our analysis showed no significant difference in serious adverse reactions in patients with severe COVID-19 compared to SOC or placebo among the eight treatment regimens (Fig. S5). Hyperglycemia is one of the common side effects of glucocorticoid, and was reported in 6 RCTs [13, 21, 28, 31, 32, 35] (*n* = 919), there regimens include HD-dm, MD-dm, VHD-dm, placebo, HD-mp. Similarly, we did not find that glucocorticoid use increased the incidence of hyperglycemia (Fig. S5).

## Discussion

Although glucocorticoids are commonly prescribed for SARS [8] and MERS [9], the efficacy of using glucocorticoids to treat COVID-19 patients remains controversial. The largest clinical trial evidence to date has shown that dexamethasone at a medium-dose (6 mg/day) reduces 28-day mortality in patients with COVID-19. However, the merits and disadvantages of other doses and types of glucocorticoids for COVID-19 treatment have not been fully explored.

This network meta-analysis was based on 19 randomized controlled trials, involving 10,544 COVID-19 patients randomly assigned to nine glucocorticoids or to placebo groups. Similar to the previous meta-analysis [38, 39], a medium-dose of dexamethasone (6 mg/day) did reduce 28-day all-cause mortality, length of hospitalization, and the need for mechanical ventilation in patients with COVID-19. We further found that very high-dose methylprednisolone (80-200 mg/day) not only reduces the above outcomes, but also has better efficacy than dexamethasone (6 mg/day).

The use of pulse therapy methylprednisolone was only reported in one RCT [34]. The analysis showed that pulse therapy methylprednisolone was better than any other dose and type of glucocorticoid, including very high-dose methylprednisolone methylprednisolone, in reducing patient’s death within 28 days. However, the duration of mechanical ventilation use and duration of ICU admission were not reported in Edalatifard et al.’s study, therefore it could not be compared with other glucocorticoid protocols.

Due to the following limitations, this network meta-analysis should be interpreted with caution. First, SARS-Cov-2 is a highly variable virus, the time span of RCTs included in our study was 2 years, during which different RCTs may enroll patients with different virus subspecies. Different virus subspecies may have different virulence and different clinical symptoms. However, the RCTs included in this network meta-analysis did not report the subspecies of virus patients were infected with, which may be a potential source of bias [40, 41]. Second, our study was conducted at the study level and may not reflect variables at the patient level, limited by the quantity and quality of the included article, further studies are needed to determine the optimal type and dosage of glucocorticoids, and to take these results into account with long-term clinical efficacy and safety to provide a basis for clinical use. Third, not all glucocorticoid treatment regimens reported the outcomes we wanted to explore. For example, pulse therapy methylprednisolone did not report the length of hospital stay, invasive ventilation utilization, and ICU admission that we were interested in.

Despite these limitations, our study has two key advantages. We divided glucocorticoid treatment regimens into 9 groups, further revealing the role of glucocorticoid type and dose in the prognosis of COVID-19 patients. Secondly, we only included randomized controlled trials on glucocorticoid therapy for COVID-19, the number of included articles was larger than the previous meta-analysis, therefore, the results were more credible.

In conclusion, all included glucocorticoid regimens were superior to placebo in reducing 28-day mortality, and methylprednisolone and medium or high-dose dexamethasone were significantly superior to other treatments, among which pulse therapy methylprednisolone was the best. In terms of length of hospital stay, glucocorticoids were superior to placebo except for unreported glucocorticoid regimens and high-dose methylprednisolone, and methylprednisolone was the best. In terms of mechanical ventilation utilization, methylprednisolone (80-200 mg/day), hydrocortisone (120-400 mg/day), dexamethasone (1.125-6 mg/day) can reduce the probability of mechanical ventilation. The sequence from high to low that glucocorticoids reduced ICU admission was: high-dose dexamethasone; medium-dose dexamethasone; very high-dose methylprednisolone; very high-dose dexamethasone; high-dose methylprednisolone, but there was no statistical significance. In terms of adverse effects, glucocorticoid use did not increase the occurrence of adverse reactions.

Different regimens of glucocorticoids have variable pleiotropic effects in the treatment of COVID-19. In order to better interpret our conclusions, we had discussed commonly used clinical dose of the above glucocorticoids in the treatment of COVID-19, the most common dosage of dexamethasone was medium dose, and the common dosage of methylprednisolone and hydrocortisone were both high dose. Their primary and secondary outcomes in the treatment of COVID-19 were: only medium dose dexamethasone can both reduce the 28-day all-cause mortality, hospitalization duration and mechanical ventilation requirement of patients, but could not improve ICU admission rate; high dose methylprednisolone was not reported in terms of mechanical ventilation requirement, there was no significant improvement in the other three outcomes. No RCTs had been reported on hospitalization duration and ICU admission in high dose hydrocortisone, and it didn’t improve 28-day all-cause mortality and mechanical ventilation requirement in COVID-19 patients.

To compare the effects of different types of glucocorticoids on the primary and secondary outcomes of the treatment of COVID-19 at the equivalent dose, we took the most commonly used glucocorticoid regimen as an example: medium dose dexamethasone, and other equivalent doses of glucocorticoids were: medium dose methylprednisolone and hydrocortisone, the type of glucocorticoids with the best performance was medium dose dexamethasone, which can significantly reduce the 28-day all-cause mortality and other secondary outcomes, including hospitalization duration and mechanical ventilation requirement of patients. While no RCTs have been conducted on methylprednisolone at this dosage till the literature retrieval was completed in this meta-analysis. As for medium dose hydrocortisone, it was only reported in the 28-day all-cause mortality and had no improvement on it.

From the above point of view, we can conclude that medium dose dexamethasone was the most commonly used glucocorticoid regimen for the treatment of COVID-19, and it has the best effect among the commonly used and equivalent doses of other glucocorticoids. The reason for the different results of the same equivalent dose of glucocorticoids used in the treatment of COVID-19 is still unclear, and we speculate that it may be due to the different types of glucocorticoids have different metabolism and half-life: dexamethasone is a long-acting glucocorticoids, methylprednisolone is a medium-acting glucocorticoids, and hydrocortisone is a short-acting glucocorticoids.

### Supplementary Information

**Additional file 1: Figure S1.** Network plot of different glucocorticoid regimens. **Figure S2.** Comparison-correction funnel plot. **Figure S3.** The results of the network meta-analysis. **Figure S4.** SUCRA ranking charts of different regimen of glucocorticoid. **Figure S5.** Forest plot of different glucocorticoid regimens.

**Additional file 2.** PRISMA 2020 Checklist.

## Data Availability

The datasets used during the current study are available from the corresponding author upon reasonable request.
