# Supplementary material for: Efficacy and safety of glucocorticoids use in patients with COVID-19: a systematic review and network meta‑analysis
Source: BMC Infect Dis. 2023 Dec 20;23:896. doi: 10.1186/s12879-023-08874-w (PMC10734118; doi:10.1186/s12879-023-08874-w)
Supplement: Supplementary file 1 — Additional file 1: Figure S1. Network plot of different glucocorticoid regimens. Figure S2. Comparison-correction funnel plot. Figure S3. The results of the network meta-analysis. Figure S4. SUCRA ranking charts of different regimen of glucocorticoid. Figure S5. Forest plot of different glucocorticoid regimens. [file 12879_2023_8874_MOESM1_ESM.docx]

## Search Strategy

Search databases: Pubmed, Web of Science, Cochrane Central Register of Controlled Trials (CENTRAL), CNKI full-text database, Wanfang Database

**Pubmed**

#1 ((("COVID-19"[Mesh]) OR (((((((((((((((((((((((((((((((COVID 19[Title/Abstract]) OR (SARS-CoV-2 Infection[Title/Abstract])) OR (Infection, SARS-CoV-2[Title/Abstract])) OR (SARS CoV 2 Infection*[Title/Abstract])) OR (2019 Novel Coronavirus Disease[Title/Abstract])) OR (2019 Novel Coronavirus Infection[Title/Abstract])) OR (2019-nCoV Disease[Title/Abstract])) OR (2019 nCoV Disease*[Title/Abstract])) OR (Disease, 2019-nCoV[Title/Abstract])) OR (COVID-19 Virus Infection*[Title/Abstract])) OR (COVID 19 Virus Infection[Title/Abstract])) OR (Infection, COVID-19 Virus[Title/Abstract])) OR (Virus Infection, COVID-19[Title/Abstract])) OR (Coronavirus Disease 2019[Title/Abstract])) OR (Disease 2019, Coronavirus[Title/Abstract])) OR (Coronavirus Disease-19[Title/Abstract])) OR (Coronavirus Disease 19[Title/Abstract])) OR (Severe Acute Respiratory Syndrome Coronavirus 2 Infection[Title/Abstract])) OR (SARS Coronavirus 2 Infection[Title/Abstract])) OR (COVID-19 Virus Disease*[Title/Abstract])) OR (COVID 19 Virus Disease[Title/Abstract])) OR (Disease, COVID-19 Virus[Title/Abstract])) OR (Virus Disease, COVID-19[Title/Abstract])) OR (2019-nCoV Infection*[Title/Abstract])) OR (2019 nCoV Infection[Title/Abstract])) OR (Infection, 2019-nCoV[Title/Abstract])) OR (COVID19[Title/Abstract])) OR (COVID-19 Pandemic[Title/Abstract])) OR (COVID 19 Pandemic[Title/Abstract])) OR (Pandemic, COVID-19[Title/Abstract])) OR (COVID-19 Pandemics[Title/Abstract]))) OR (("SARS-CoV-2"[Mesh]) OR ((((((((((((((((((((SARS Coronavirus 2[Title/Abstract]) OR (Coronavirus 2, SARS[Title/Abstract])) OR (Coronavirus Disease 2019 Virus[Title/Abstract])) OR (2019 Novel Coronavirus*[Title/Abstract])) OR (Coronavirus, 2019 Novel[Title/Abstract])) OR (Novel Coronavirus, 2019[Title/Abstract])) OR (Wuhan Seafood Market Pneumonia Virus[Title/Abstract])) OR (SARS-CoV-2 Virus*[Title/Abstract])) OR (SARS CoV 2 Virus[Title/Abstract])) OR (Virus, SARS-CoV-2[Title/Abstract])) OR (2019-nCoV[Title/Abstract])) OR (COVID-19 Virus*[Title/Abstract])) OR (COVID 19 Virus[Title/Abstract])) OR (Virus, COVID-19[Title/Abstract])) OR (Wuhan Coronavirus[Title/Abstract])) OR (Coronavirus, Wuhan[Title/Abstract])) OR (COVID19 Virus*[Title/Abstract])) OR (Virus, COVID19[Title/Abstract])) OR (Viruses, COVID19[Title/Abstract])) OR (Severe Acute Respiratory Syndrome Coronavirus 2[Title/Abstract]))))

#2 (((("Glucocorticoids"[Mesh]) OR (((((Glucocorticoid[Title/Abstract]) OR (Glucocorticoid Effect[Title/Abstract])) OR (Effect, Glucocorticoid[Title/Abstract])) OR (Glucorticoid Effects[Title/Abstract])) OR (Effects, Glucorticoid[Title/Abstract]))) OR (("Steroids"[Mesh]) OR (((Steroid[Title/Abstract]) OR (Catatoxic Steroids[Title/Abstract])) OR (Steroids, Catatoxic[Title/Abstract])))) OR (((((((("Dexamethasone"[Mesh]) OR "Betamethasone"[Mesh]) OR "Prednisone"[Mesh]) OR "Prednisolone"[Mesh]) OR "Methylprednisolone"[Mesh]) OR "Hydrocortisone"[Mesh]) OR "Cortisone"[Mesh]) OR (triamcinolon[Title/Abstract])))

#3 #1 AND #2 Filters: Randomized Controlled Trial

**WOS（22.04.29）**

#1 TS=(“COVID-19” OR “COVID 19” OR “SARS-CoV-2 Infection” OR “Infection, SARS-CoV-2” OR “SARS CoV 2 Infection” OR “SARS-CoV-2 Infections” OR “2019 Novel Coronavirus Disease” OR “2019 Novel Coronavirus Infection” OR “2019-nCoV Disease*” OR “2019 nCoV Disease” OR “Disease, 2019-nCoV” OR “COVID-19 Virus Infection*” OR “COVID 19 Virus Infection” OR “Infection, COVID-19 Virus” OR “Virus Infection, COVID-19” OR “Coronavirus Disease 2019” OR “Disease 2019, Coronavirus” OR “Coronavirus Disease-19” OR “Coronavirus Disease 19” OR “Severe Acute Respiratory Syndrome Coronavirus 2 Infection” OR “SARS Coronavirus 2 Infection” OR “COVID-19 Virus Disease*” OR “COVID 19 Virus Disease” OR “Disease, COVID-19 Virus” OR “Virus Disease, COVID-19” OR “2019-nCoV Infection*” OR “2019 nCoV Infection” OR “Infection, 2019-nCoV” OR “COVID19” OR “COVID-19 Pandemic” OR “COVID 19 Pandemic” OR “Pandemic, COVID-19” OR “COVID-19 Pandemics” OR “SARS-CoV-2” OR “SARS Coronavirus 2” OR “Coronavirus 2, SARS” OR “Coronavirus Disease 2019 Virus” OR “2019 Novel Coronavirus*” OR “Coronavirus, 2019 Novel” OR “Novel Coronavirus, 2019” OR “Wuhan Seafood Market Pneumonia Virus” OR “SARS-CoV-2 Virus*” OR “SARS CoV 2 Virus” OR “Virus, SARS-CoV-2” OR “2019-nCoV” OR “COVID-19 Virus*” OR “COVID 19 Virus” OR “Virus, COVID-19” OR “Wuhan Coronavirus” OR “Coronavirus, Wuhan” OR “COVID19 Virus*” OR “Virus*, COVID19” OR “Severe Acute Respiratory Syndrome Coronavirus 2”)

#2 Ts=(“Glucocorticoids” OR “Glucocorticoid” OR “Glucocorticoid Effect” OR “Effect, Glucocorticoid” OR “Glucorticoid Effects” OR “Effects, Glucorticoid” OR “Steroids” OR “Steroid” OR “Catatoxic Steroids” OR “Steroids, Catatoxic” OR “Adrenal Cortex Hormones” OR “Hormones, Adrenal Cortex” OR “Corticosteroids” OR “Corticosteroid” OR “Corticoids” OR “Corticoid” OR “Adrenal Cortex Hormone” OR “Cortex Hormone, Adrenal” OR “Hormone, Adrenal Cortex” OR Dexamethasone OR Betamethasone OR Prednisone OR prednisolone OR methylprednisolone OR triamcinolone OR Dehydrocortisone OR Hydrocortisone OR cortisone)

#3 TS= clinical trial* OR TS=research design OR TS=comparative stud* OR TS=evaluation stud* OR TS=controlled trial* OR TS=follow-up stud* OR TS=prospective stud* OR TS=random* OR TS=placebo* OR TS=(single blind*) OR TS=(double blind*)

#4 #1 AND #2 AND #3

**Cochrane**

#1 Mesh descriptor: [COVID-19] explode all trees

#2 Mesh descriptor: [Glucocorticoid] explode all trees

#3 #1 AND #2

**CBMdisc**

#1 "Glucocorticoid/ Administration and dosage "

#2 "COVID-19/ pharmacotherapy "

#1 AND #2

**CNKI database**

#1（Title：Glucocorticoid）OR（Title：Steroid）OR（Title：Cortisol hormone）

#2（Title：COVID-19）OR（Title, key words, abstract：COVID-19）OR（Title, key words, abstract：SARS-Cov-2）

#3 #1 AND #2

**WANFANG database**

#1 Title or key words:(COVID-19) OR Title or key words:(COVID-19) OR Title or key words:( SARS-Cov-2)

#2 Title or key words:(Glucocorticoid) OR Title or key words:( Steroid) OR Title or key words:( Cortisol hormone)

#3 #1 AND #2

## Inclusion Criteria

1. aged 18 years or older

2. hospitalized with confirmed SARS-CoV-2 infection

3. be able to sign informed consent

## Exclusion Criteria

1. severe immunosuppression (human immunodeficiency virus infection and long-term use of immunosuppressive agents),

2. pregnant or breastfeeding women,

3. corticosteroid needed for other diseases,

4. any contraindications of steroid administration

5. uncontrolled diabetes mellitus

6. uncontrolled hypertension

7. secondary bacterial or fungal infection

8. unwilling or unable to participate or complete the study

9. participation in other RCTs.

10. lack of willingness to participate in the study.


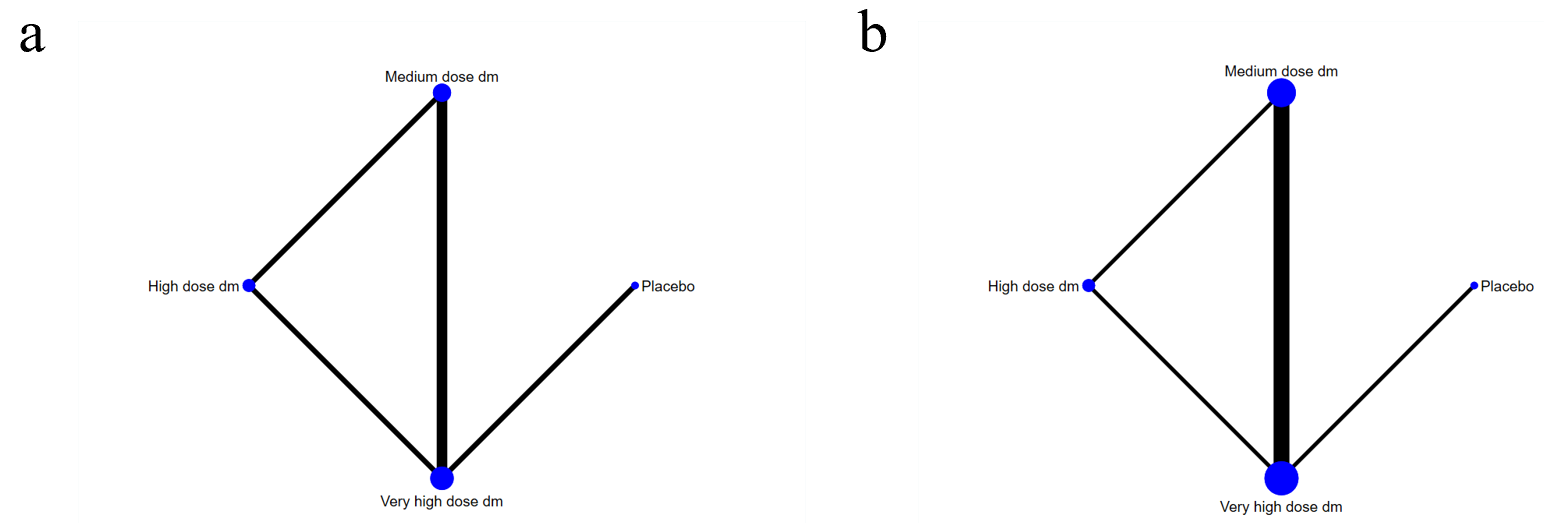


Figure S1. Network plot of different glucocorticoid regimens.

(a)Mechanical ventilation duration; (b) ICU duration.


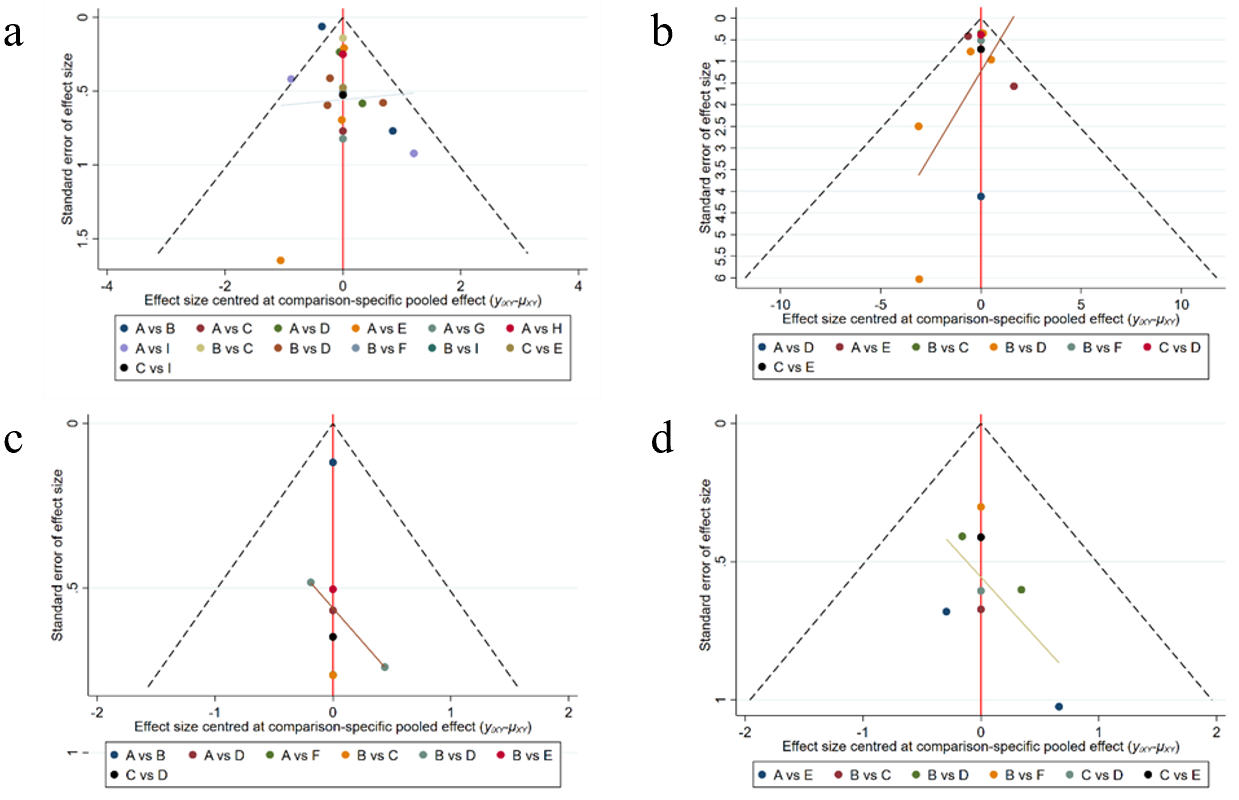


Figure S2. Comparison-correction funnel plot.

(A) 28-day all-cause mortality; (B) Hospitalization duration; (C) Mechanical ventilation requirement; (D) ICU admission.

A represents placebo, B represents medium-dose dexamethasone; C represents high-dose dexamethasone; D represents very high-dose dexamethasone; E represents high-dose methylprednisolone; F represents very high-dose methylprednisolone; G represents pulse therapy methylprednisolone; H represents medium-dose hydrocortisone; I represents high-dose hydrocortisone.


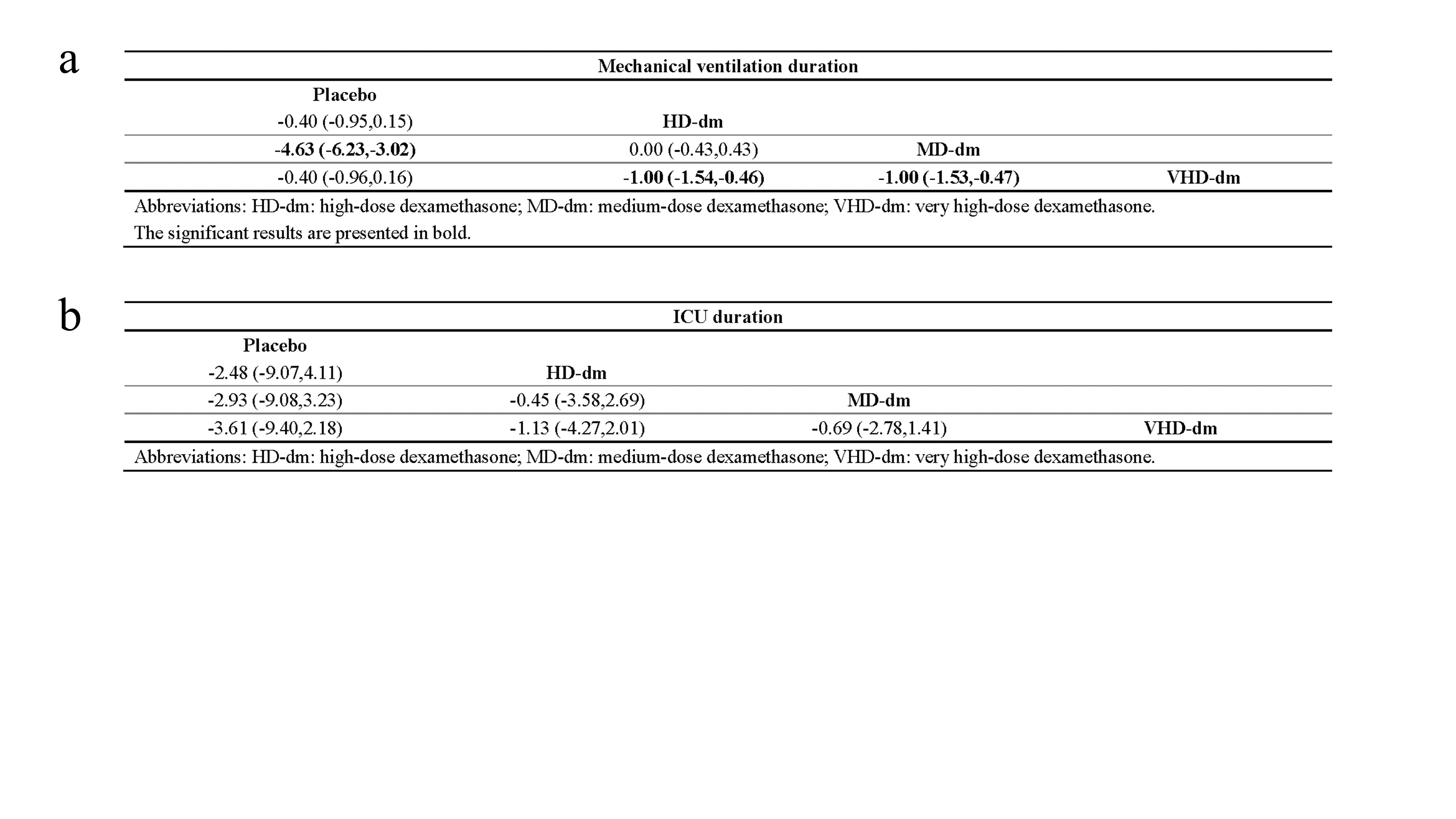


Figure S3 The results of the network meta-analysis.

(a)Mechanical ventilation duration; (b) ICU duration.


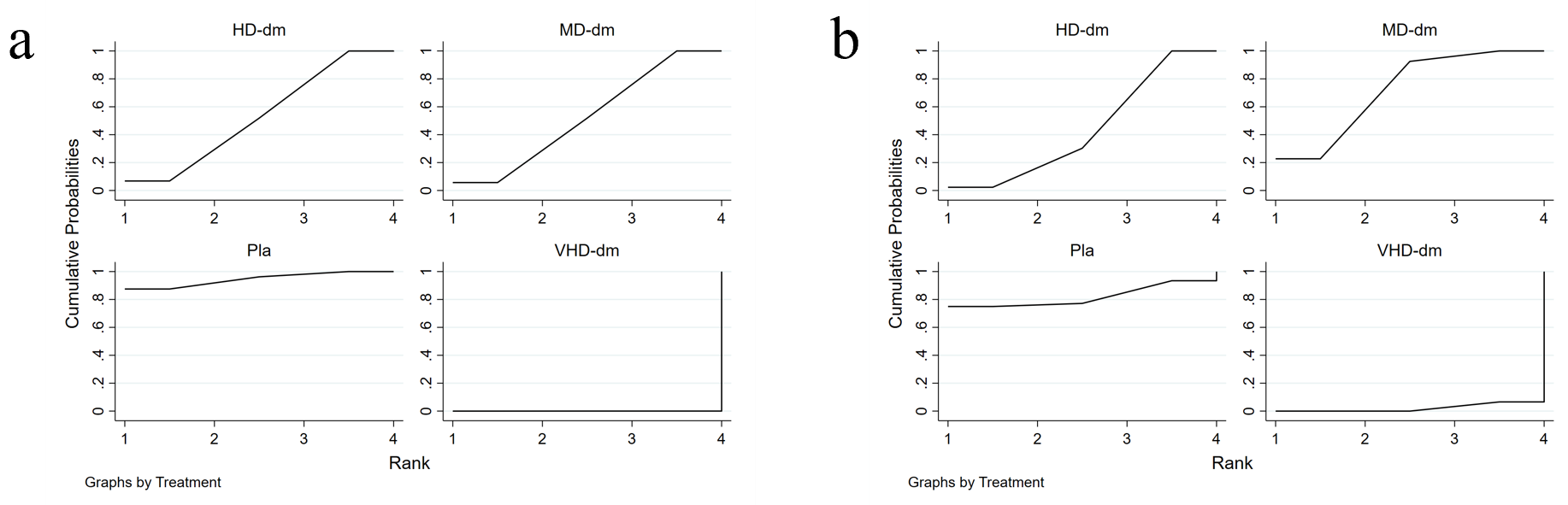


Figure S4. SUCRA ranking charts of different regimen of glucocorticoid.

(a)Mechanical ventilation duration; (b) ICU duration.


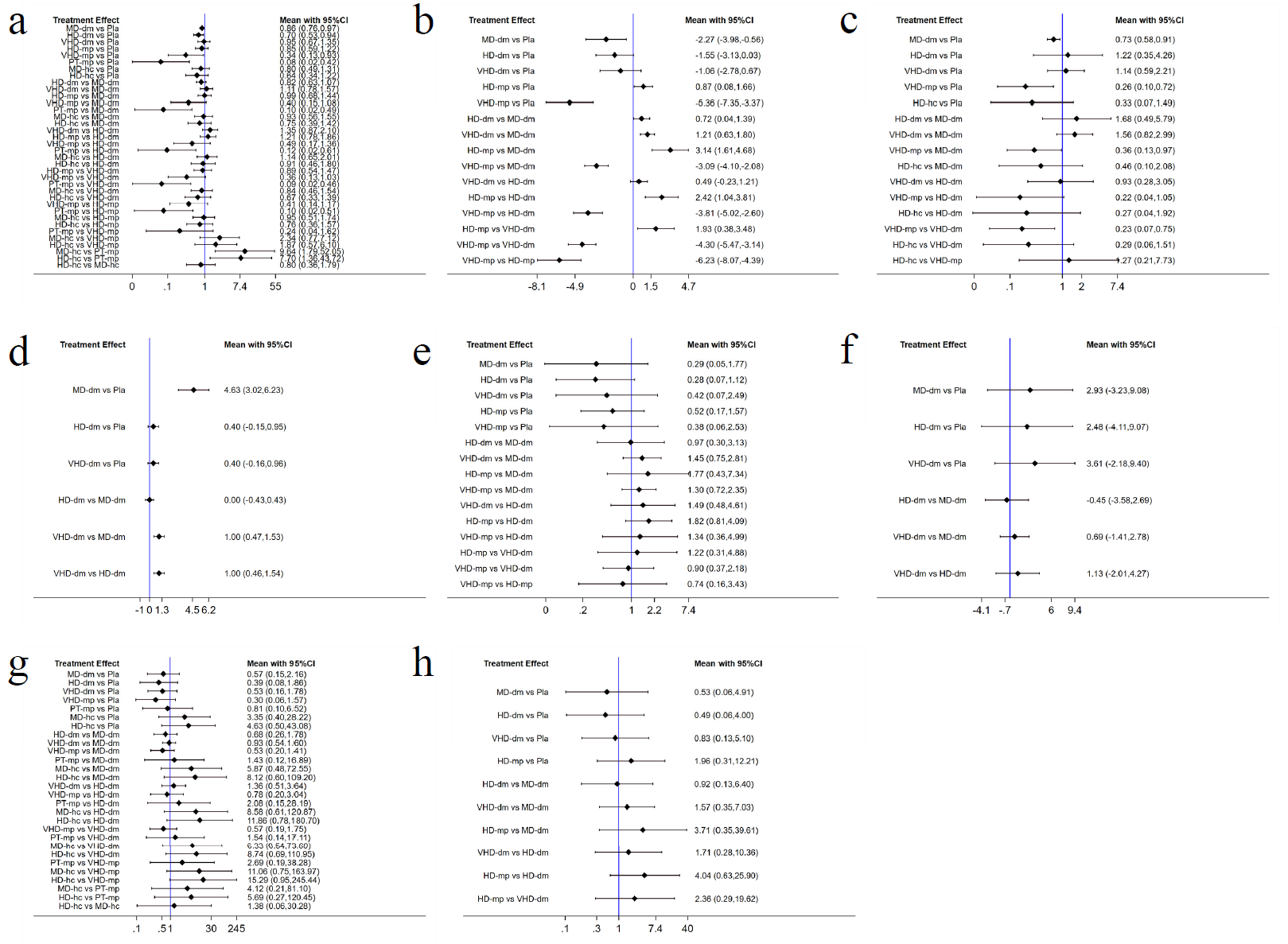


Figure S5. Forest plot of different glucocorticoid regimens

(a) 28-day all-cause mortality; (b) Hospitalization duration; (c)Mechanical ventilation requirement; (d)Mechanical ventilation duration; (e) ICU admission. (f) ICU duration; (g) Serious adverse effects. (h) Hyperglycemia.
